# Dose-Dense Epirubicin and Cyclophosphamide Followed by Docetaxel as Adjuvant Chemotherapy in Node-Positive Breast Cancer

**DOI:** 10.1155/2013/404396

**Published:** 2013-09-25

**Authors:** Hamid Reza Mirzaei, Parisa Sabet Rasekh, Fatemeh Nasrollahi, Parto Sabet Rasekh, Zahra Akbari Tirabad, Hamid Reza Moein, Taban Ghaffari Pour, Parastoo Hajian

**Affiliations:** Department of Radiation & Oncology, Cancer Research Center, Shohadaye-Tajrish Hospital of Shahid Beheshti University of Medical Sciences and Health Services, Tajrish Square, Tehran, Iran

## Abstract

*Background*. Adding taxanes to anthracycline-based adjuvant chemotherapy has shown significant improvement particularly in node-positive patients, but optimal dose and schedule remain undetermined. *Objectives*. This study aimed to assess the feasibility of dose-dense epirubicin and cyclophosphamide followed by docetaxel in node-positive breast cancer. *Methods*. All Patients first received 4 cycles of epirubicin (100 mg/m^2^) and cyclophosphamide (600 mg/m^2^) at 2-week interval then followed by docetaxel (100 mg/m^2^) at 2-week interval for 4 cycles, with daily Pegfilgrastim (G-CSF) that was administered in all patients on days 3–10 after each cycle of epirubicin and cyclophosphamide infusion. *Results*. Fifty-eight patients with axillary lymph node-positive breast cancer were enrolled in the study, of whom 42 (72.4%) completed the regimen. There were two toxicity-related deaths, one patient due to grade 4 febrile neutropenia and the other due to congestive heart failure. Grade 3/4 neutropenia and febrile neutropenia were 13.8% and 5.1%. The most common grade 3/4 nonhematological complications were as follows: skin-nail disorders (48.3%), hand-foot syndrome (34.4%), paresthesia (38%), arthralgia (27.5%), and paresis (24.1%). *Conclusions*. Dose-dense epirubicin and cyclophosphamide followed by docetaxel with G-CSF support are not feasible, and it is not recommended for further investigation.

## 1. Introduction

Adjuvant chemotherapy substantially reduces the risk of recurrence and death among women with breast cancer [1, 2]. Anthracyclines and taxanes are mainstays in treating women with axillary node-positive breast cancer, and anthracycline-containing regimens have been shown to confer higher response rates and longer overall survival [3]. Large adjuvant taxane trials were designed to test whether there was a benefit to taxanes after, or combined with, anthracycline-containing regimens [4–6].

Taxanes given sequentially or concurrently with anthracyclines have resulted in a significant improvement in disease-free and overall survival particularly in node-positive disease [4–7].

The semisynthetic taxoid docetaxel (Taxotere) is probably the most active single agent in breast cancer, and results in advanced disease supported the development of trials including both paclitaxel and docetaxel, in combination or in sequence with anthracyclines, in the adjuvant setting [8]. 

Clinical evidence suggested that docetaxel was a more effective taxane than paclitaxel [9]. Another study demonstrated that concurrent administration of docetaxel with doxorubicin and cyclophosphamide was more effective than fluorouracil, doxorubicin, and cyclophosphamide which led to regulatory approval of docetaxel for node-positive breast cancer [10].

Despite growing evidence [11–14] that adding a taxane to conventional anthracycline-based regimens is one of the best therapeutic options for patients with node-positive disease [15] and taxanes are already approved in this indication, the optimal dose and schedule of taxane remain undetermined.

Hryniuk and Levine, 1986, first suggested that dose-dense adjuvant chemotherapy was correlated to disease-free survival in breast cancer [14]. Dose-intensity refers to administration of drugs with a shortened treatment interval. Based on mathematical modeling of tumor growth [16, 17], shortening the interval between treatment cycles from every 3 weeks to 2 weeks, or using a dose-dense schedule, improved the outcome for patients with breast cancer with axillary node-positive disease [18].

A randomized trial has demonstrated a significant survival for women, with positive lymph node breast cancer, whom are treated with dose-dense (2-week intervals) chemotherapy accompanied by granulocyte colony stimulating factor (G-CSF) support compared to conventional 3-week schedules [19].

Epirubicin is an anthracycline that may be less cardiotoxic and less myelosuppressive than doxorubicin, with no less of antitumor efficacy. The major adverse effects of epirubicin are acute dose-limiting haematological toxicity and cumulative dose-related cardiac toxicity. These effects are less severe after epirubicin administration than after equimolar doses of doxorubicin. The equimolar dose ratios of doxorubicin to epirubicin for myelosuppression and cardiotoxicity are 1 : 1.2 and 1 : 1.7–2.0, respectively [20, 21].

Based on these findings and because of limited such studies in our country, we evaluated the safety and feasibility of dose-dense epirubicin and cyclophosphamide (EC) followed by docetaxel (T) with G-CSF support in women with node-positive breast cancer.

## 2. Materials and Methods

### 2.1. Patient Eligibility

Women eligible for the study were between 18 and 70 years of age and had undergone primary surgery (i.e., mastectomy, tumorectomy, or lumpectomy), with histologically proven invasive breast cancer and at least one histologically resected positive auxiliary lymph node (size of tumor was assessed during surgery by pathological examination). Eastern Cooperative Oncology Group performance 0-1, adequate biological functions (hemoglobin >10 g/dL; absolute neutrophil count >1.5 × 10^9^/L; platelets >100 × 10^9^/L; serum creatinine clearance >60 mL/min; bilirubin < upper normal limit (UNL); alkaline phosphatase (ALP) <5 × UNL and aminotransferases <2.5 × UNL), and normal cardiac function were confirmed by clinical examination and left ventricular ejection fraction (LVEF >50%). The hormone receptor assays were performed by immunohistochemistry (IHC) in all cases and a positive test defined by ≥1%.

Patients were excluded if they had even one of the following: T4 stage (clinical and pathological), inflammatory breast cancer, ductal carcinoma in situ (DCIS), basal skin carcinoma, in situ cervical caner or anticancer therapy, other serious illness or medical conditions (most notably cardiac and/or neurologic disorders), sensory or motor neuropathy of severity greater than WHO grade 1, pregnant or breast-feeding patient or inadequate contraception, or any other condition that was considered to make the patient ineligible for this study by the investigators. The study was performed in accordance with the declaration of Helsinki, and written informed consent was obtained prior to participation in the study. The study protocol was reviewed and approved by institutional review board of the Shohadaye-Tajrish Hospital.

### 2.2. Patient Assessment

Eligible patients who had given a consent were invited to attend the assessment to provide baseline data as follows: full medical history and physical examination, hematology and biochemistry assessment (such as renal and liver function tests), hormone receptor status, chest radiography and/or computed tomography (CT) scan, electrocardiogram and echocardiography, abdominal and pelvic ultrasound or computed tomography (CT) scan, bone scan, and other evaluation based on symptoms of patients.

### 2.3. Treatment Plan

All patients received epirubicin (100 mg per square meter of body-surface area, given by slow intravenous push during a period of 5 to 15 minutes) and cyclophosphamide (600 mg per square meter by intravenous infusion for 30 to 60 minutes) every 2 weeks for four cycles followed by docetaxel (100 mg per square meter by intravenous infusion for 60 minutes) every 2 weeks for four cycles; granulocyte-colony-stimulating factor (G-CSF) 300 microgram daily was administered in all patients on days 3–10 of each course of epirubicin and cyclophosphamide. 

Premedication for EC consisted of a 5-HT3 serotonin receptor antagonist (e.g., granisetron 3 mg or ondansetron 1 mg) and dexamethasone 16 mg intravenously. Standard premedication with glucocorticoids, H1 and H2 receptor blockers (e.g., promethazine, clemastine, and ranitidine), was given before docetaxel administration. Actual body weight was used for body surface area calculations. A complete blood count with leukocyte differential was performed before each chemotherapy treatment. Patients were seen every two weeks during treatment for history and physical examination and assessment of performance status and toxicity. 

### 2.4. Dose Modification

Treatment was given on day 1 of every cycle if absolute neutrophils count (ANS) and platelet were ≥1.5 × 10^9^/L and ≥100 × 10^9^/L, respectively, or grade 4 nonhematologic toxicities (excluding nausea, vomiting, and alopesia) day 1 doses in the subsequent cycle were reduced, but doses in the current cycle were administered according to protocol. A maximum of two-dose reduction was allowed. When platelet count, absolute neutrophil count, or failure of nonhematologic toxicities to recover to ≤ grade 1 were noted, either singly or in combination, on the scheduled start day of the subsequent cycle, treatment was delayed by up to one week, and complete blood count and toxicity grading were repeated weekly. Patients requiring a treatment delay of more than three weeks were removed from the study. 

After chemotherapy completed, radiation therapy in patients treated with conservation was conducted following the last cycle of chemotherapy and after recovery from any toxicity according to standard institutional dosing guidelines and techniques. Patients whose tumors expressed either (or both) the estrogen or progesterone receptor positive were offered a 5-year course of tamoxifen 20 mg/day. Postmenopausal patients were offered aromatase inhibitors as an alternative to tamoxifen. 

### 2.5. Assessment of Treatment

Toxicity for each cycle was assessed before the commencement of the following cycle and was graded using the National Cancer Institute Common Toxicity Criteria (NCI-CTC version 3).

Patients were followed closely by history and physical examination within 15 days and one month after last infusion, and this followup was processed every 2-month intervals for the first year of chemotherapy completion then every six-month interval for years 4-5. Each visit included a complete blood count, along with hematologic studies and chemistries (liver and renal function tests), chest X-ray, and ECG. Computed tomography scan of the chest, abdomen, and pelvic and a bone scan (or both) were considered if clinically indicated by symptomatology or abnormal laboratory values at the discretion of the physician. After treatment completed, echocardiography was carried out in all patients. Mammography was performed on the remaining breast(s) annually.

### 2.6. Statistical Analysis

The objective of the study was to evaluate the toxicity of epirubicin and cyclophosphamide plus docetaxel in node-positive breast cancer. Fifty-eight eligible patients were required to answer the trial aim. Descriptive methods were applied for all the variables. Statistical analyses were carried out using SPSS software version 16, and *P* < 0.05 was considered significant.

The end point was the incidence (*r*) of grade 3/4 toxicity. The study was designed as a one-stage three-outcome phase II study, in which H0 was: *r* > 50% and HA: *r* < 25%. Under these assumptions and with *α* and *β* errors rate of 5% each, 58 patients were assigned to reject a toxic treatment (with >50% grade 4) and accept a nontoxic treatment (with <25% grade 4) with a probability >90%. If <14 grade 4 toxic events occurred, the treatment was to be considered tolerable. If >29 grade 4 toxic events occurred, the treatment was to be considered intolerable. If 15–28 grade 4 toxic events occurred, the study was not conclusive. 

## 3. Results

### 3.1. Patient Characteristics

Fifty-eight eligible patients were enrolled into the study from April 2007 to March 2009 in Shohadaye-Tajrish Hospital. Patients' characteristics at the time of entering the study are listed in Table 1.

The mean ±SD age of the patients was 44.52 ± 9.65 year, and 68.9% of the patients were <50 years old. The median number of examined lymph nodes was 12 (range 5–24), and mean number of involved lymph nodes was 4. Median tumor size was 3 cm.

The tumor was positive for estrogen receptor in 63.8% of patients, positive for progesterone receptor in 65.5%, positive for both estrogen and progesterone receptors in 60%, and positive for HER2 in 39.7%. 

### 3.2. Toxicity

The chemotherapy cycle was completed in all patients except in sixteen patients (27.6%). Thirty-two patients (55%) have undergone any grade 4 adverse events that fifteen of them went off the study after the second or third infusion of docetaxel cycle due to grade 4 skin-nail disorders concomitant with myalgia, arthralgia, and neuropathy which was not tolerable by patients and did not further receive docetaxel. Also, grade 4 toxicity occurred after last infusion of docetaxel in eight patients. Treatment was delayed in 33 patients (62.1%). The cause of delay was nausea, vomiting, diarrhea, skin-nail disorders, and neuropathy.

Two patients died due to toxicity, one patient because of grade 4 febrile neutropenia after the third infusion of docetaxel cycle and another patient due to congestive heart failure after the last infusion of docetaxel. 

Seventeen patients (29.3%) were hospitalized due to adverse events: one patient because of grade 4 febrile neutropenia, thirteen patients due to grade 4 adverse events such as skin-nail disorders, arthralgia, dehydration, nausea, vomiting, and diarrhea, and three patients due to reduction of LVEF and signs of congestive heart failure in which one of them developed to heart failure and in others subsequently recovered. 

Initial echocardiography in all patients was normal, but at the end of study, two patients (3.4%) had abnormal echocardiography, and they experienced congestive heart failure which was reversible. Also, 50 patients (86.2%) experienced total alopecia. 

As a consequence of the assessment of blood counts, there was a nearly high rate of grade 1/2 neutropenia and febrile neutropenia, but it was asymptomatic and almost did not modify the treatment plan; however, grade 3/4 of these adverse events was uncommon. However, it was the reason for the use of G-CSF in all patients. Albeit none of the patients suffered grade 3/4 thrombocytopenia, grade 3 anemia was 6.9%, and grade 1/2 anemia was common (58.6%). 

Incidences of hematological and nonhematological toxicities are shown in Table 2.

At the time of the analysis, the median follow-up period was 20 months; four systemic relapses were observed, and two patients died during follow-up period due to brain metastasis. 

## 4. Discussion

As a therapeutic option for patients with node-positive breast cancer, the combination of taxane with an anthracycline has been recently investigated in several studies [12, 13, 22, 23]. Some studies included that dose-dense regimens (2-week intervals) not only prolong both disease-free survival and overall survival but also are as safe and as tolerated as giving the 3-week intervals conventional regimens [18, 24].

This study evaluated the toxicity of dose-dense epirubicin and cyclophosphamide followed by docetaxel with G-CSF support in node-positive breast cancer. It was found that this chemotherapy regimen was not tolerable, with a high incidence of grade 3/4 toxicities. 

Although in our trial there was one death due to grade 4 febrile neutropenia, frequency of grade 4 hematologic toxicity was low, and it is likely that this low incidence was due to Jones et al. [24] compared accelerated epirubicin or doxorubicin with cyclophosphamide given at 2-week interval with G-CSF support with 3-week intervals in early breast cancer and observed fewer grade 3/4 neutropenia.

Citron et al. [18] compared standard 3 weekly and accelerated 2 weekly schedules of concurrent doxorubicin and cyclophosphamide followed by paclitaxel, or sequential doxorubicin, paclitaxel, and cyclophosphamide. They found that grade 4 neutropenia was more frequent in the standard 3 weekly schedules than in the accelerated regimens (33% versus 6%, *P* < 0.0001). 

In our study, three patients (5/2%) experienced signs of congestive heart failure and one developed to heart failure and death. Piedbois et al. [22] compared docetaxel, epirubicin, and cyclophosphamide every 3 weeks with dose-dense 2-weekly schedules of epirubicin and cyclophosphamide followed by docetaxel or the reverse sequence. As in our study, they found that the most frequent hematologic toxicity was neutropenia; also it was more frequent in group receiving epirubicin and cyclophosphamide followed by docetaxel, and frequency of grade 3-4 cardiac toxicity was 3% in group receiving dose-dense regimen of epirubicin and cyclophosphamide followed by docetaxel in comparison with no cases of this toxicity in another schedules. They concluded that no significant difference was between nonhematological toxicity and between the standard and dose-dense schedules of doxorubicin or epirubicin with cyclophosphamide. 

In another phase II trial of docetaxel plus epirubicin, grade 3-4 cardiac disorders occurred in two patients [25]. Whereas in other trials of epirubicin and docetaxel, very low level of cardiotoxicity (reversible congestive heart failure in one of sixty patients only) or no grade 3-4 cardiac toxicity was observed [18, 24–26]. 

Recently, Sparano reported a randomized trial of four cycles of AC every 3 weeks postoperatively and followed by one of four taxane-based treatments, namely, paclitaxel administered weekly or every three weeks, or docetaxel administered weekly or every three weeks. Results showed that in comparison with the group receiving standard therapy, the group receiving weekly paclitaxel had significantly more moderate-to-severe neuropathy, and the group receiving docetaxel every 3 weeks had significantly more severe neutropenia and its associated complications. Besides, it was more frequent with docetaxel than paclitaxel. The arms that received weekly paclitaxel and 3-weekly docetaxel were associated with improved disease-free survival compared with 3-weekly paclitaxel, and the disease-free survival of the weekly docetaxel arm was similar to 3-weekly paclitaxel. The weekly paclitaxel arm was also associated with improved survival compared with 3-weekly paclitaxel, and 5-year overall survival was higher with weekly paclitaxel.

We found that a significant fraction of patients had grade 3 or higher of skin disorders and peripheral neuropathy during treatment, and also nearly a high percentage of patients experienced moderate-to-severe arthralgia that were treated symptomatically. 

Our trial was consistent with a study that demonstrated more patients given dose-dense regimens reported nail disorders, hand-foot syndrome, peripheral neuropathy and fluid retention of any grade, and more grade 3 or 4 events, compared with standard group [18, 23–27].

Another phase II of epirubicin plus docetaxel versus 5-fluorouracil plus epirubicin and cyclophosphamide concluded that skin infection and peripheral neuropathy were more frequent in docetaxel group [25]. These toxic effects, albeit manageable, notably impaired patients' quality of life, and the benefit/risk ratio of dose-dense regimens in the adjuvant setting should be cautiously evaluated.

Various studies demonstrated that docetaxel with doxorubicin and cyclophosphamide (AC) or with epirubicin and cyclophosphamide (EC) have had more favorable chemotherapy results, and epirubicin is an anthracycline that may be less cardiotoxic than doxorubicin [20].

Albeit, a randomized pilot phase II study of AC and EC signified no significant difference between AC and EC cardiac toxicities, unless some toxicities, such as sepsis neutropenia, stomatitis, nausea, and vomiting, were more frequent in AC regimen in comparison with EC, but EC regimen was more tolerable than AC [22]. However, incidence of toxicity in our study was remarkably higher. Followup of patients in this study will demonstrate results for the efficacy of this treatment, which is a secondary endpoint of disease-free and overall survival.

The end point was the incidence of grade 4 toxic events. According to the statistical design of the trial, 32 patients (55%) experienced grade 4 toxicity with this chemotherapy regimen. The study therefore cannot conclude that this regimen has an acceptable safety profile. Furthermore, two toxic deaths and sixteen related adverse events which led to treatment discontinuation, leading to assume that dose-dense epirubicin and cyclophosphamide plus docetaxel with G-CSF support is not feasible and a tolerable regimen, and this regimen might not be selected for further assessment in other trials. In addition, this result is in agreement with several previous publications that have reported nonfeasibility of dose-dense docetaxel mainly due to hand-foot-skin toxicity but also severe problems with nail disorders and peripheral neuropathy [22, 28, 29].

In conclusion, our results indicate that the dose-dense epirubicin and cyclophosphamide followed by docetaxel with G-CSF support was not tolerable and feasible and also might not be selected for further assessment in phase III trials according to the protocol hypothesis. 

## Figures and Tables

**Table 1:** Clinical characteristic of patients.

Characteristic	Mean ± SD
Age (years)	44.52 ± 9.65
Pathological tumor size (cm)	4.32 ± 2.79
No. of node analyze	11.48 ± 6.39
No. of positive nodes	4.47 ± 4.29

	No. (%)

Side involved	
Right	26 (44.8)
Left	32 (55.2)
Histology	
Ductal	52 (89.7)
Lobular	4 (6.9)
Others	2 (3.4)
Hormone receptors	
ER	
Positive	37 (63.8)
Negative	21 (36.2)
PR	
Positive	38 (65.5)
Negative	20 (34.5)
HER-2	
Positive	23 (39.7)
Negative	35 (60.3)
Hormone therapy	
Positive	45 (77.6)
Negative	13 (22.4)
Regimen of hormone therapy	
Tamoxifen	40 (88.9)
Others	5 (11.1)

**Table 2:** Incidence of toxicities in treated patients.

Toxicities	Normal no. (%)	G1,2 no. (%)	G3 no. (%)	G4 no. (%)
Haematological toxicity				
Neutropenia	34 (58.6)	16 (27.6)	8 (13.8)	—
Febrile neutropenia	40 (69)	15 (25.9)	2 (3.4)	1 (1.7)
Anemia	20 (34.5)	34 (58.6)	4 (6.9)	—
Thrombocytopenia	50 (86.2)	8 (13.8)	—	—
Nonhaematological toxicity				
Skin and nail disorders	4 (6.9)	26 (44.8)	11 (19)	17 (29.3)
Scaling	16 (27.6)	25 (43.1)	9 (15.5)	8 (13.8)
Stomatitis	30 (51.7)	20 (34.5)	6 (10.3)	2 (3.4)
Hand-foot syndrom	23 (39.7)	15 (25.9)	6 (10.3)	14 (24.1)
Erythema	48 (82.8)	10 (17.2)	—	—
Paresis	27 (46.6)	17 (29.3)	10 (17.2)	4 (6.9)
Paresthesia	20 (34.4)	16 (27.6)	19 (32.8)	3 (5.2)
Myalgia	41 (70.7)	11 (19)	4 (6.9)	2 (3.4)
Arthralgia	23 (39.7)	19 (32.8)	13 (22.4)	3 (5.1)
Nausea	6 (10.3)	36 (62.1)	10 (17.2)	6 (10.3)
Vomiting	21 (36.2)	25 (43.1)	7 (12.1)	5 (8.6)
Fluid retention	38 (65.5)	18 (31.1)	2 (3.4)	—
Phlebitis	51 (87.9)	4 (6.9)	3 (5.2)	—
Any grade 4 event		32 (55%)		
Death		2 (3.4)		
